# Altered expression of cell cycle and apoptotic proteins in chronic hepatitis C virus infection

**DOI:** 10.1186/1471-2180-8-133

**Published:** 2008-08-05

**Authors:** Saira Sarfraz, Saeed Hamid, Anwar Siddiqui, Snawar Hussain, Shahid Pervez, Graeme Alexander

**Affiliations:** 1 Department of Biological and Biomedical Sciences, Aga Khan University, Karachi, Pakistan; 2 Department of Medicine, Aga Khan University, Karachi, Pakistan; 3 Department of Microbiology and Immunology, Loyola University Medical Center, Maywood, Illinios, USA; 4 Department of Histopathology, Aga Khan University Hospital, Karachi, Pakistan; 5 Department of Medicine, Addenbrooke's Hospital, University of Cambridge, Cambridge, CB2 2QQ, UK

## Abstract

**Background:**

A disrupted cell cycle progression of hepatocytes was reported in chronic hepatitis C virus (HCV) infection, which can contribute significantly in the associated pathogenesis. The present study aimed to further elaborate these disruptions by evaluating the expression of key cell cycle and apoptotic proteins in chronic HCV infection with particular reference to genotype 3. Archival liver biopsy specimens of chronic HCV-infection (n = 46) and normal histology (n = 5) were analyzed by immunohistochemistry using antibodies against proliferation marker Mcm-2, G1 phase marker Cyclin D1, S phase marker Cyclin A, cell cycle regulators p21 (CDK inhibitor) and p53 (tumor suppressor protein), apoptotic protein Caspase-3 and anti-apoptotic protein Bcl-2.

**Results:**

Elevated Mcm-2 expression was observed in hepatocytes in chronic HCV infection, indicating increased cell cycle entry. Cyclin D1 expression was higher than cyclin A, which suggests a slow progression through the G1 phase. Expression of cell cycle regulators p21 and p53 was elevated, with no concordance between their expressions. The Mcm-2 and p21 expressions were associated with the fibrosis stage (p = 0.0001 and 0.001 respectively) and that of p53 with the inflammation grade (p = 0.051). Apoptotic marker, Caspase-3, was mostly confined to sinusoidal lining cells with little expression in hepatocytes. Anti-apoptotic protein, Bcl-2, was negligible in hepatocytes and detected principally in infiltrating lymphocytes. Expression of all these proteins was unrelated to the HCV genotype and were detected only rarely in the hepatocytes of normal liver.

**Conclusion:**

The results showed an arrested cell cycle state in the hepatocytes of chronic HCV infection, regardless of any association with genotype 3. Cell cycle arrest is characterized by an increased expression of p21, in relation to fibrosis, and of p53 in relation to inflammation. Furthermore, expression of p21 was independent of the p53 expression and coincided with the reduced expression of apoptotic protein Caspase-3 in hepatocytes. The altered expression of these cell cycle proteins in hepatocytes is suggestive of an impaired cell cycle progression that could limit the regenerative response of the liver to ongoing injury, leading to the progression of disease.

## Background

Hepatitis C virus (HCV) infections account for a vast majority of viral hepatitis cases in some geographical areas. In Pakistan, around 6% of people are estimated to be infected with HCV [1]. These figures are alarming, since patients currently asymptomatic with relatively mild disease will eventually progress to the end-stage liver disease and develop hepatocellular carcinoma (HCC). Currently, there is no vaccine against HCV and antiviral treatment is not only expensive but relatively toxic and is sufficiently ineffective in treating all of the patients [2]. This underscores the need for more effective therapies. A better understanding of the molecular mechanisms underlying the pathology of chronic HCV infections could be helpful in identifying novel therapeutic targets against this disease.

The hallmarks of chronic HCV infection in the liver are inflammation, necrosis, hepatocellular damage and fibrosis. The damage caused by inflammation and necrosis leads generally to proliferation of the remaining hepatocytes, a characteristic of liver regeneration [3]. Proliferative responses of hepatocytes to HCV infection are particularly important in subsequent pathogenesis as hepatocytes are the primary site of HCV replication and receive different cellular stresses from lymphocytes and Kupffer cells. Several studies have measured proliferative activity in liver tissue from patients with chronic HCV infection using a variety of markers such as Ki-67, proliferating cell nuclear antigen (PCNA) and mini-chromosome maintenance protein-2 (Mcm-2) [4-6]. Among these, Mcm-2 has been documented as a more sensitive proliferation marker than Ki-67 in chronic HCV-infected patients [5].

The molecular events during proliferation are related closely to the cell cycle and its regulation. When stimulated to proliferate, hepatocytes first enter the G1 phase of the cell division cycle which is followed by DNA synthesis, or the S phase. Progression through each phase of the cell cycle involves periodic activation of phase-specific protein kinase complexes comprising of cyclins and cyclin dependent kinases (CDKs). Therefore, cyclin D-CDK4/CDK6 complex is activated in the G1 phase and cyclin A-CDK2 is activated in the S phase [7,8]. Cyclin-CDK complexes are known to be regulated negatively by CDK inhibitors (CKIs), which are induced in response to different stimuli including DNA damage and oxidative stress. One such inhibitor is the p21^WAF1/CIP1 ^(p21) protein that binds to various cyclin-CDK complexes and inhibits the activity of CDK in both p53-dependent and p53-independent fashion [9,10]. Both p53 and p21 may be of relevance while studying the HCV induced disease mechanism, as a number of viral proteins modulate p53 and p21 expression and activity *in vitro *[11,12]. Besides a role in cell cycle regulation, p53 also activates and represses various genes involved in apoptosis, including the gene for pro-apoptotic protein Bax and anti-apoptotic protein Bcl-2 [13].

A number of *ex vivo *studies revealed that the expression of HCV proteins in cultured cells modulates normal cell cycle regulation and apoptosis [14,15]. Similarly, studies of liver specimens from patients with chronic HCV infection have also shown impaired hepatocyte proliferation [16] and enhanced apoptosis [17] that may play a role in the subsequent pathogenesis. A recent study has shown marked differences in the phase-distribution of cycling hepatocytes using immunohistochemical staining [18], with a striking reduction in the markers of late cell cycle phases (i.e. the S phase and beyond) consistent with G1 arrest. Moreover, impaired proliferation of hepatocytes has been implicated in the development of cirrhosis [19], suggesting the involvement of cell cycle machinery in liver disease progression. Evaluation of the expression of key proteins related to the cell cycle and apoptosis in chronically infected patients with HCV would be of significance to understand disease pathogenesis, and will help in identifying novel prognostic indicators.

In the present study, we aimed to evaluate the expression of cell cycle and apoptotic proteins in chronic HCV-infected patients. The majority (69%) of patients included in our study were infected by HCV genotype-3 (GT-3). Such patients are known to have a high prevalence of steatosis [20], which might influence the hepatocyte replication pathway. Therefore, we examined whether the proliferation and cell cycle progression of hepatocytes is different in GT-3 infected patients as compared to other genotypes. The results indicate an arrested cell cycle state in hepatocytes, as revealed by the reduced expression of S phase cyclin and the increased expression of the G1 inhibitor p21. Further, no association between the expression of cell cycle regulator p21 and that of p53 was found. Finally, the analysis of apoptosis related proteins Caspase-3 and anti-apoptotic Bcl-2 expression in the same set of biopsies revealed minimal expression in hepatocytes.

## Results

### Expression of Mcm-2 and cyclins in hepatocytes

**Figure 1:**
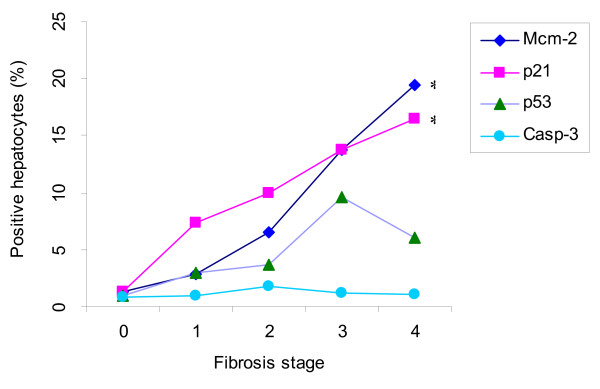
**Hepatocyte expression of cell cycle proteins and apoptotic marker in chronic hepatitis C patients with respect to fibrosis stage**. * = p-value ≤ 0.05.

The proliferative potential of hepatocytes was examined by immunostaining for Mcm-2 that identifies replicating cells as well as cells that are arrested in the cell cycle [21]. Increased expression of Mcm-2 was observed in HCV-infected liver tissues (see Additional file 1, median (range) = 11% (1.5 – 29.6)) with no significant difference between the genotype 3 and others (1 and 4). In normal liver tissue its expression was less than 0.01%. Interestingly, a significant increase in Mcm-2 expression was found with the progression of the fibrosis stage (Figure 1, Jonckheere-Terpstra test, p = 0.0001).

Analysis of cell cycle phase markers in patients with chronic HCV infection revealed that the proportion of Mcm-2 hepatocytes expressing cyclin D1 (G1 phase marker) was 35% (3 – 54), which was significantly higher than that for cyclin A (S phase marker) (4.0% (0.1 – 18), Mann-Whitney U test, p = 0.0001). In normal liver tissues, Cyclin D and A expression was minimal.

### Expression of cell cycle regulators p21 and p53

**Figure 2:**
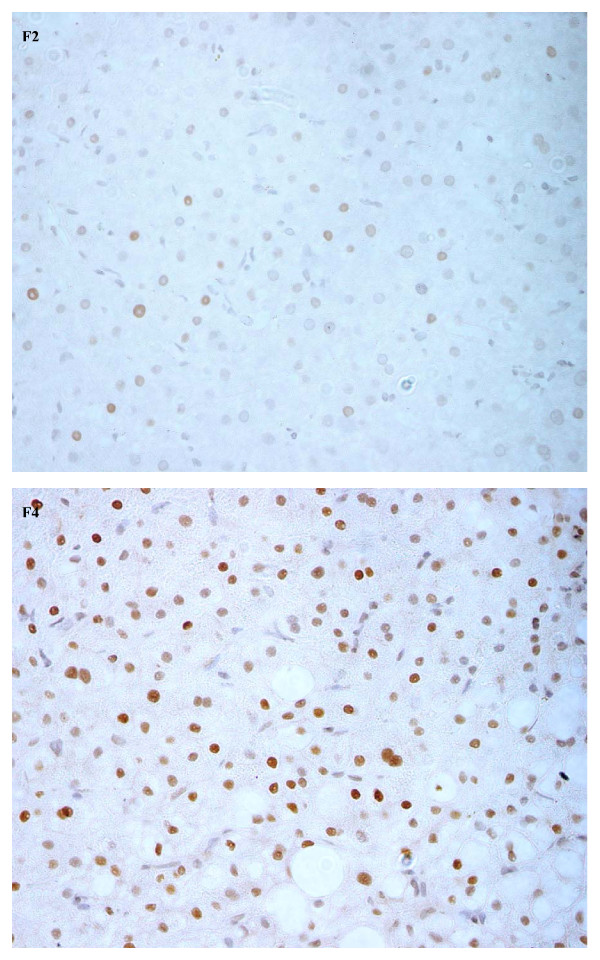
**Expression of cell cycle regulator p21**. Nuclear expression of p21 in hepatocytes of chronic hepatitis C patients, fibrosis stage 2 (F2) and 4 (F4) (Mayer hematoxylin, magnification 400×).

**Figure 3:**
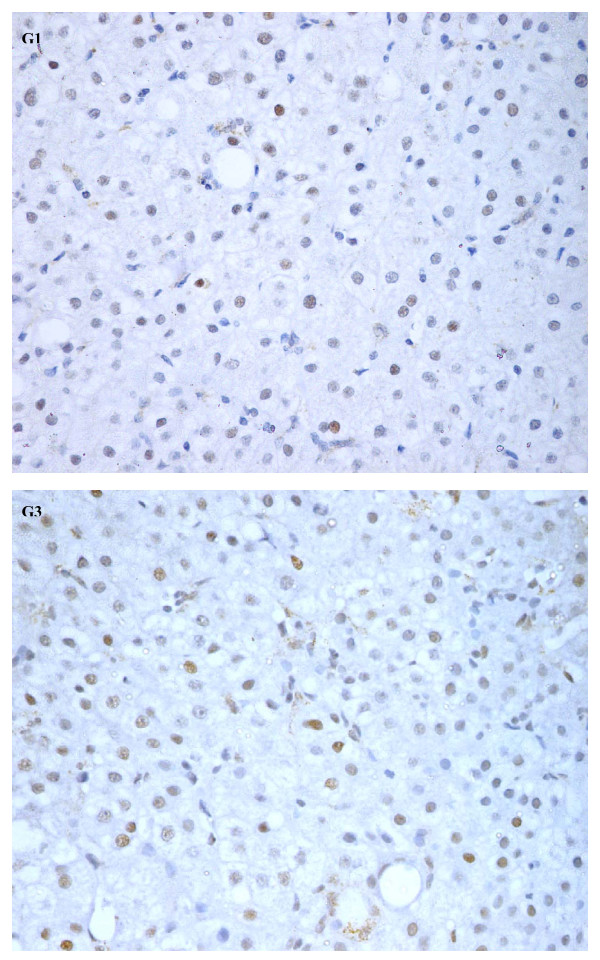
**Expression of cell cycle regulator p53**. Nuclear p53 expression in liver biopsy specimens from chronic hepatitis C patient, grade 1 (G1) and 3 (G3) (Mayer hematoxylin, magnification 400×).

In normal liver tissues very low expression (< 0.01%) of cell cycle regulator p21 was observed, whereas 78% (36/46) of patients with chronic HCV infection showed increased expression (Figure 2) with a median (range) of 9.3% (1.0 – 19.5). p53 was also expressed rarely (< 0.01%) in normal liver tissues. In comparison, 52% (24/46) of patients with chronic HCV infection had enhanced expression of p53 (Figure 3) with a median (range) of 8.5% (1.0 – 15.6). Of these, 33% (15/46) expressed both p53 and p21, with no correlation between their expression (Spearman rank correlation, p = 0.38). p21 positive hepatocyte expression correlated with progression of the fibrosis stages (Figure 1, Jonckheere Terpstra, p = 0.001) but not with the inflammation grade. In contrast, p53 expression was found to be associated weakly with the inflammation grade (Jonckheere Terpstra test, p = 0.051), but not with the fibrosis stage. No significant association was found between these regulators and the degree of steatosis. Similarly, no difference in their expression levels was noted in GT-3 patients when compared to patients infected with other genotypes.

### Expression of apoptosis proteins

**Figure 4:**
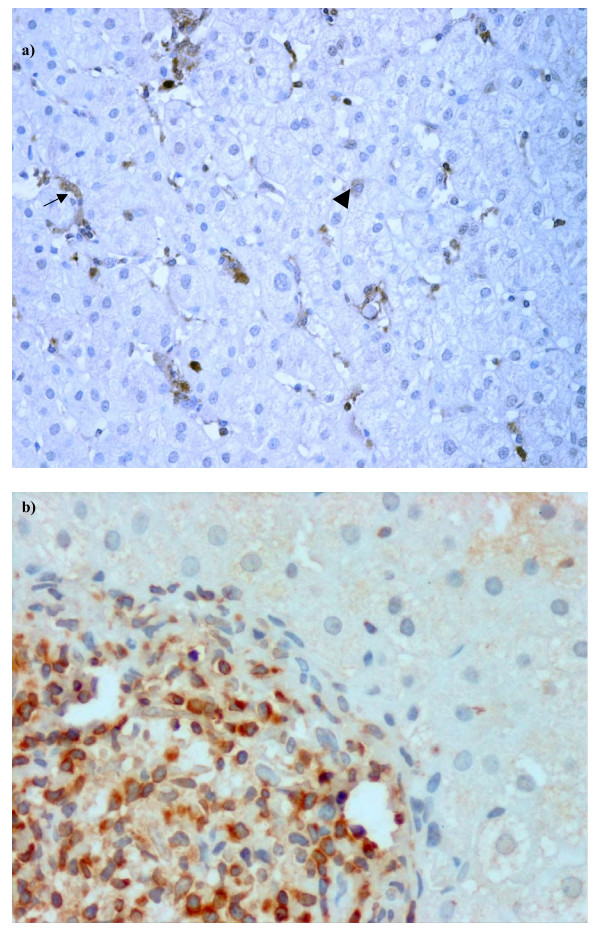
**Apoptotic activity in hepatocytes of Hepatitis C patients**. Immunostaining in liver biopsies of chronic hepatitis C patient (HCV). a) Caspase-3 expression was observed principally in sinusoidal lining cells (arrow) and rarely in hepatocytes (arrow head), b) Bcl-2 staining observed in inflammatory cells, (magnification 400×).

Expression of the two apoptotic proteins Caspase-3 and Bcl-2, which are involved in apoptosis execution and anti-apoptotic functions, respectively, were assessed. Caspase-3 was detected in 43% of the patients, and was expressed principally within sinusoidal lining cells, including endothelial cells and Kupffer cells (Figure 4a). In hepatocytes its expression was relatively low, with a median (range) of 1.8% (0.1–3.2), while no correlation was found between Caspase-3 and cell cycle regulators or with any of the histological features.

Expression of Bcl-2 was either negative or negligible in the hepatocytes of HCV-infected patients, but was more apparent in other cell types including inflammatory cells and sinusoidal lymphocytes (Figure 4b). In normal liver tissues, both caspase-3 and Bcl-2 were rarely detected.

## Discussion

In the present study, we have examined the expression of cell cycle proteins with particular reference to HCV GT-3. Our results provide evidence of p21-mediated hepatocyte cell cycle arrest in chronic HCV-infected patients regardless of any association with GT-3. A previous report by Marshall et al has also shown G1 arrest in hepatocytes using the same cell cycle markers in a different set of patients [18]. We broadened the focus of our study by providing indirect evidence that p21 expression was independent of p53 expression. Furthermore, minimal expression of the apoptosis related protein Caspase-3 was observed in hepatocytes which were without any detectable expression of anti-apoptotic Bcl-2.

In the initial phases of chronic liver disease, hepatocytes proliferate to restore liver mass and maintain hepatic function [22,23]. Consistent with this principle, we found an increased number of hepatocytes expressing the cell proliferation marker, Mcm-2. However, the ongoing inflammation and injury causes disruptions in cell cycle progression. These disruptions are evidenced in the present study by decreased expression of S phase cyclin (Cyclin A) and increased expression of p21. Moreover, the expression of these proteins did not differ significantly in genotype 3 infected patients when compared to other genotypes. This indicates that HCV GT-3 has no peculiar effect on hepatocyte cell cycle disruption, though a significant difference is observed in its response to the treatment and development of steatosis [20]. Nevertheless, it can not be neglected that this comparison was made on a small group of patients and further studies to analyze the relevance of the HCV genotype with cell cycle disruption are needed.

Expression of both p21 and Mcm-2 proteins in hepatocytes showed a positive association with the progression of fibrosis. This brings into focus the relationship of hepatocyte proliferation and cell cycle arrest with changes taking place in the extra cellular matrix. It also supports previous observations reported by Clouston et al which suggested that hepatocyte replicative arrest drives the bile ductular reaction and fibrosis in chronic HCV infected patients [24].

Although the association of p21 with hepatocyte replicative arrest and disease activity has been described in chronic hepatitis C patients [18,24], it is not known as to whether this increase is due to up-regulation of p53 or some other mechanism. We investigated this aspect by evaluating p53 expression in the same set of biopsies and found that both p53 and p21 co-expressed in only 33% [1/3] of the patients, but their expression did not correlate with each other. This indicates that p21 expression in hepatocytes during chronic HCV infection is probably mediated through p53-independent mechanisms involving TGF-β [10] and oxidative stress [25]. On the other hand, HCV might also be involved in manipulating the expression of these cell cycle regulators, as observed *in vitro *following ectopic expression of viral proteins [11,26]. Nonetheless, direct evidence of these virus-induced manipulations in chronic HCV infection is still lacking in literature.

In normal conditions p53 expression remains undetectable by immunohistochemistry. However, exposure to a variety of cellular stresses leads either to an increased synthesis rate of wild type p53 or the production of a mutated p53 with increased stability that would become detectable by immunohistochemistry. We observed an accumulation of p53 in the hepatocytes of chronic HCV patients with a significant percentage in the advanced stages of fibrosis. This is consistent with a previous report by Papakyriakou et al [27] that has shown p53 expression in severe viral hepatitis. But whether this accumulation of p53 is due to the increased synthesis or the production of mutated p53 is not yet determined. We also observed an association of p53 expression with a grade of inflammation that was marginally significant (p = 0.051). Accumulation of p53 in response to inflammatory stress [28] as well as it's contribution to inflammation-induced injury is also noted in published data [29,30]. This could be due to the increased production of reactive oxygen species during inflammation that can directly cause DNA damage [31] and thus p53 activation [32].

Since hepatocyte proliferation plays an important role in the regenerative response to hepatic damage, a replicative arrest in hepatocytes could, therefore, impair the regenerative response of the liver to the ongoing injury. In fact, experiments in knock out mice have shown a significant contribution of the p53/p21 system in the impairment of liver regeneration following partial hepatectomy [33]. This in turn contributes to the pathogenesis of cirrhosis and hepatocellular carcinoma. Moreover, impaired cell cycle kinetics could also lead to the activation of the apoptosis pathway to preserve cell viability and genetic integrity. Studies on hepatic biopsies have shown a variable degree of apoptosis in HCV infected patients ranging from 0.54%–20%, depending on the method used [34]. Some of these studies have also shown a correlation of apoptosis with liver pathology, but the mechanisms involved were not well defined. In our present study, we analyzed the apoptotic activity by examining the expression of Caspase-3, a downstream effector molecule in the apoptotic pathway. Caspase-3 has been shown to be involved in p21 cleavage and the subsequent translocation to cytoplasm in a p53-dependent manner [35]. Our results revealed a minor expression of Caspase-3 in hepatocytes, while the p21 antigen was apparent in the nucleus of hepatocytes. Based on these observations, we contemplate that the increased expression of p21 in hepatocytes is probably responsible for the reduced expression of Caspase-3 in hepatocytes. The inhibitory role of p21 in the apoptotic pathway has been documented previously by several investigators [36,37]. On the other hand, our results differ with a previous report by Bental et al which showed high Caspase-3 expression in the hepatocytes of chronic hepatitis C patients [38]. Possible explanations for this discrepancy could be the different antibodies and sample size used, along with the high prevalence of genotype 3 infected patients in our study. We further examined if the low apoptotic index in hepatocytes is due to the high levels of Bcl-2. Interestingly, Bcl-2 expression was mainly observed in mononuclear/inflammatory cells but not in hepatocytes, suggesting that the reduced apoptosis in hepatocytes is not due to the anti-apoptotic activity of Bcl-2.

## Conclusion

Our analysis of the cell cycle and apoptotic proteins during chronic HCV infection has shown an impaired proliferation of hepatocytes, which is characterized by increased expression of p21 and p53. Furthermore, increased nuclear expression of p21 in hepatocytes coincides with the minimal expression of the apoptotic protein Caspase-3 and the anti-apoptotic Bcl2. In the scenario of HCV-associated liver inflammation, an altered expression of these cell cycle proteins will probably reduce the number of replicating hepatocytes that can limit the regenerative response of the liver. The present work therefore, offers a broader aspect to understand the mechanisms involved in the pathogenesis of HCV infection.

## Methods

### Liver biopsy samples and histology

After obtaining approval from the ethical review committee of Aga Khan University, paraffin-embedded liver biopsy specimens were studied from forty-six untreated patients with chronic HCV infection. All patients were HCV antibody positive, as determined by a commercially available enzyme-linked immunosorbent assay kit (Abbott Laboratories, Chicago, IL). Patients having other etiologies for chronic liver disease including HBV infection were not included in the study. Histopathological grading and staging was performed on formalin-fixed tissue samples by an experienced liver pathologist according to the Batts and Ludwig scoring system [39] at the Histopathology laboratory of Aga Khan University. Steatosis was assessed by the same pathologist using the Brunt scoring system [40]. The demographic features of the patients are given in Table 1. Liver biopsy specimens from five control patients were also included in the study. These were selected on the basis of a normal histology as determined by light microscopy. None had evidence of acute or chronic liver disease and all were negative for both anti-HCV antibodies and HCV-RNA.

**Table 1:** Demographic and clinical features of the patients

No. of patients	46
Age (years)	42+9.3
Gender (male)	77%
Viral genotype	
3	63.0%
Other genotypes (1 or 4)	19.6%
*NA	17.4%
Stage of Fibrosis (0–4), n	
0	9
1	10
2	13
3	7
4	7
Grade of Inflammation, (0–4), n	
0	6
1	8
2	17
3	15
Steatosis, n	
None	25
Mild	6
Moderate	9
Severe	4

*Not available

### Antibodies

Mouse monoclonal antibodies to cyclin D1 (DSC-6, 1:40), p21 (cyclin dependent kinase inhibitor, SX-118, 1:40), p53 (tumor suppressor protein, DO-7, 1:40) and Bcl-2 (anti-apoptotic protein, Klon-124, 1:50) were purchased from DAKO (Denmark). Caspase-3 (apoptotic marker, E-8, 1:100) was purchased from Santa Cruz (CA) and cyclin A (6E6, 1:50) from Novacastra (MA). The Mcm-2 antibody (CRCT2.1, 1:50) was a gift from Abcam (UK).

### Immunohistochemistry

For immunohistochemistry 4-μm sections of paraffin-embedded liver tissues were de-paraffinized and rehydrated using a xylene-alcohol sequential wash. Following this, all sections were subjected to antigen retrieval by heating in 0.01 M citrate buffer (pH 6.0) in a pressure-cooker. This was followed by endogenous peroxidase ablation using 0.6% hydrogen peroxide in methanol. After blocking with 10% goat serum for two hours, the sections were incubated with primary antibodies overnight at 4°C. The slides were subsequently incubated, first with biotinylated secondary antibodies and then with the labeled ABC (streptavidin horseradish peroxidase detection system, DAKO) using diaminobenzidine (Sigma) as a substrate. Finally, the slides were counterstained with haematoxylin and mounted in DPEX for examination. Positive controls were included in each batch of the slides and negative controls were obtained by omitting the primary antibodies.

### Quantitative analysis

The immuno-stained sections were examined using a Nikon Eclipse E8000 and an image analysis system, (Nikon, Japan). Evaluations of Mcm-2, cyclin D, cyclin A, p21 and p53 expression were based on nuclear staining; Caspase-3 expression was assessed by both cytoplasmic and nuclear staining while Bcl-2 expression was assessed by membranous staining. Quantification of positive hepatocytes was undertaken by two independent observers by counting approximately 1000 hepatocytes at magnification 400× in five randomly selected fields. The positive hepatocytes were expressed as a percentage of the total cells counted in each case and values for the subsequent analysis were obtained from the mean of the two independent observations. In the case of Mcm-2, p21, p53 and Caspase-3 this was defined as the labeling index (LI). For cyclin D and cyclin A, positive hepatocytes were expressed as a percentage of the number of hepatocytes expressing Mcm-2 for each case and defined as the labeling fractions (LF) [18].

### Statistical Analysis

Data analyses were performed using the Statistical Package for Social Sciences (SPSS, 14.0). Associations between the expression of Mcm-2, p21, p53 or Caspase-3 with the histological features (stage of fibrosis, grade of inflammation and steatosis) were analyzed using the Jonckheere-Terpstra test. To determine the differences in the expression of cell cycle proteins between genotype groups, the Mann-Whitney U test was used. Correlations among cyclin D1 – cyclin A and p21 – p53 were evaluated by the Spearman's rank correlation coefficient. A p-value of ≤ 0.05 was considered significant.

## Authors' contributions

SS conducted the experimental work, formulated the data and wrote the first draft of the manuscript. SHa formulated the idea, supervised the research protocol and the development of the manuscript. AS supervised the work and the writing of the manuscript. SP supervised immunohistochemistry. SHu was involved in setting up initial experiments and defining the research protocol. GA helped in the formulation of the concepts and the review of the manuscript.

## Supplementary Material

Additional file 1 **Expression of Mcm-2 and G1-S phase Cyclins**. Immunohistochemical staining of biopsy specimens from HCV-infected patients (HCV). Sections were stained using a) anti-Mcm-2, b) anti-Cyclin D and c) anti-Cyclin A. Positive hepatocytes are stained brown (Mayer hematoxylin, magnification 400×). Click here for file

## References

[B1] Hamid S, Umar M, Alam A, Siddiqui A, Qureshi H, Butt J (2004). PSG consensus statement on management of hepatitis C virus infection--2003. J Pak Med Assoc.

[B2] Pearlman BL (2004). Hepatitis C infection: a clinical review. South Med J.

[B3] Schuppan D, Krebs A, Bauer M, Hahn EG (2003). Hepatitis C and liver fibrosis. Cell Death Differ.

[B4] Farinati F, Cardin R, D'Errico A, De Maria N, Naccarato R, Cecchetto A, Grigioni W (1996). Hepatocyte proliferative activity in chronic liver damage as assessed by the monoclonal antibody MIB1 Ki67 in archival material: the role of etiology, disease activity, iron, and lipid peroxidation. Hepatology.

[B5] Freeman A, Hamid S, Morris L, Vowler S, Rushbrook S, Wight DG, Coleman N, Alexander GJ (2003). Improved detection of hepatocyte proliferation using antibody to the pre-replication complex: an association with hepatic fibrosis and viral replication in chronic hepatitis C virus infection. J Viral Hepat.

[B6] Lake-Bakaar G, Mazzoccoli V, Ruffini L (2002). Digital image analysis of the distribution of proliferating cell nuclear antigen in hepatitis C virus-related chronic hepatitis, cirrhosis, and hepatocellular carcinoma. Dig Dis Sci.

[B7] Sherr CJ (2000). The Pezcoller lecture: cancer cell cycles revisited. Cancer Res.

[B8] Malumbres M, Barbacid M (2001). To cycle or not to cycle: a critical decision in cancer. Nat Rev Cancer.

[B9] Cox LS (1997). Multiple pathways control cell growth and transformation: overlapping and independent activities of p53 and p21Cip1/WAF1/Sdi1. J Pathol.

[B10] Datto MB, Li Y, Panus JF, Howe DJ, Xiong Y, Wang XF (1995). Transforming growth factor beta induces the cyclin-dependent kinase inhibitor p21 through a p53-independent mechanism. Proc Natl Acad Sci U S A.

[B11] Nguyen H, Mudryj M, Guadalupe M, Dandekar S (2003). Hepatitis C virus core protein expression leads to biphasic regulation of the p21 cdk inhibitor and modulation of hepatocyte cell cycle. Virology.

[B12] Kwun HJ, Jung EY, Ahn JY, Lee MN, Jang KL (2001). p53-dependent transcriptional repression of p21(waf1) by hepatitis C virus NS3. J Gen Virol.

[B13] Miyashita T, Krajewski S, Krajewska M, Wang HG, Lin HK, Liebermann DA, Hoffman B, Reed JC (1994). Tumor suppressor p53 is a regulator of bcl-2 and bax gene expression *in vitro* and *in vivo*. Oncogene.

[B14] Ohkawa K, Ishida H, Nakanishi F, Hosui A, Ueda K, Takehara T, Hori M, Hayashi N (2004). Hepatitis C virus core functions as a suppressor of cyclin-dependent kinase-activating kinase and impairs cell cycle progression. J Biol Chem.

[B15] Yang XJ, Liu J, Ye L, Liao QJ, Wu JG, Gao JR, She YL, Wu ZH, Ye LB (2006). HCV NS2 protein inhibits cell proliferation and induces cell cycle arrest in the S-phase in mammalian cells through down-regulation of cyclin A expression. Virus Res.

[B16] Werling K, Szentirmay Z, Szepesi A, Schaff Z, Szalay F, Szabo Z, Telegdy L, David K, Stotz G, Tulassay Z (2001). Hepatocyte proliferation and cell cycle phase fractions in chronic viral hepatitis C by image analysis method. Eur J Gastroenterol Hepatol.

[B17] Calabrese F, Pontisso P, Pettenazzo E, Benvegnu L, Vario A, Chemello L, Alberti A, Valente M (2000). Liver cell apoptosis in chronic hepatitis C correlates with histological but not biochemical activity or serum HCV-RNA levels. Hepatology.

[B18] Marshall A, Rushbrook S, Davies SE, Morris LS, Scott IS, Vowler SL, Coleman N, Alexander G (2005). Relation between hepatocyte G1 arrest, impaired hepatic regeneration, and fibrosis in chronic hepatitis C virus infection. Gastroenterology.

[B19] Funakoshi F MT (2004). Proliferative capability of hepatocytes and expression of G1-related cell cycle molecules in the development of liver cirrhosis in rats. Intern J Mol Med.

[B20] Rubbia-Brandt L, Quadri R, Abid K, Giostra E, Male PJ, Mentha G, Spahr L, Zarski JP, Borisch B, Hadengue A, Negro F (2000). Hepatocyte steatosis is a cytopathic effect of hepatitis C virus genotype 3. J Hepatol.

[B21] Stoeber K, Tlsty TD, Happerfield L, Thomas GA, Romanov S, Bobrow L, Williams ED, Williams GH (2001). DNA replication licensing and human cell proliferation. J Cell Sci.

[B22] Callea F, Brisigotti M, Fabbretti G, Sciot R, Van Eyken P, Favret M (1991). Cirrhosis of the liver. A regenerative process. Dig Dis Sci.

[B23] Delhaye M, Louis H, Degraef C, Le Moine O, Deviere J, Peny MO, Adler M, Galand P (1999). Hepatocyte proliferative activity in human liver cirrhosis. J Hepatol.

[B24] Clouston AD, Powell EE, Walsh MJ, Richardson MM, Demetris AJ, Jonsson JR (2005). Fibrosis correlates with a ductular reaction in hepatitis C: roles of impaired replication, progenitor cells and steatosis. Hepatology.

[B25] Russo T, Zambrano N, Esposito F, Ammendola R, Cimino F, Fiscella M, Jackman J, O'Connor PM, Anderson CW, Appella E (1995). A p53-independent pathway for activation of WAF1/CIP1 expression following oxidative stress. J Biol Chem.

[B26] Arima N, Kao CY, Licht T, Padmanabhan R, Sasaguri Y, Padmanabhan R (2001). Modulation of cell growth by the hepatitis C virus nonstructural protein NS5A. J Biol Chem.

[B27] Papakyriakou P, Tzardi M, Valatas V, Kanavaros P, Karydi E, Notas G, Xidakis C, Kouroumalis E (2002). Apoptosis and apoptosis related proteins in chronic viral liver disease. Apoptosis.

[B28] Staib F, Robles AI, Varticovski L, Wang XW, Zeeberg BR, Sirotin M, Zhurkin VB, Hofseth LJ, Hussain SP, Weinstein JN, Galle PR, Harris CC (2005). The p53 tumor suppressor network is a key responder to microenvironmental components of chronic inflammatory stress. Cancer Res.

[B29] Goodman JE, Hofseth LJ, Hussain SP, Harris CC (2004). Nitric oxide and p53 in cancer-prone chronic inflammation and oxyradical overload disease. Environ Mol Mutagen.

[B30] Schafer T, Scheuer C, Roemer K, Menger MD, Vollmar B (2003). Inhibition of p53 protects liver tissue against endotoxin-induced apoptotic and necrotic cell death. Faseb J.

[B31] Choi J, Ou JH (2006). Mechanisms of liver injury. III. Oxidative stress in the pathogenesis of hepatitis C virus. Am J Physiol Gastrointest Liver Physiol.

[B32] Helton ES, Chen X (2007). p53 modulation of the DNA damage response. J Cell Biochem.

[B33] Stepniak E, Ricci R, Eferl R, Sumara G, Sumara I, Rath M, Hui L, Wagner EF (2006). c-Jun/AP-1 controls liver regeneration by repressing p53/p21 and p38 MAPK activity. Genes Dev.

[B34] Fischer R, Baumert T, Blum HE (2007). Hepatitis C virus infection and apoptosis. World J Gastroenterol.

[B35] Fan G, Ma X, Wong PY, Rodrigues CM, Steer CJ (2004). p53 dephosphorylation and p21(Cip1/Waf1) translocation correlate with caspase-3 activation in TGF-beta1-induced apoptosis of HuH-7 cells. Apoptosis.

[B36] Jin YH, Yoo KJ, Lee YH, Lee SK (2000). Caspase 3-mediated cleavage of p21WAF1/CIP1 associated with the cyclin A-cyclin-dependent kinase 2 complex is a prerequisite for apoptosis in SK-HEP-1 cells. J Biol Chem.

[B37] Levkau B, Koyama H, Raines EW, Clurman BE, Herren B, Orth K, Roberts JM, Ross R (1998). Cleavage of p21Cip1/Waf1 and p27Kip1 mediates apoptosis in endothelial cells through activation of Cdk2: role of a caspase cascade. Mol Cell.

[B38] Bantel H, Lugering A, Poremba C, Lugering N, Held J, Domschke W, Schulze-Osthoff K (2001). Caspase activation correlates with the degree of inflammatory liver injury in chronic hepatitis C virus infection. Hepatology.

[B39] Batts KP, Ludwig J (1995). Chronic hepatitis. An update on terminology and reporting. Am J Surg Pathol.

[B40] Brunt EM, Janney CG, Di Bisceglie AM, Neuschwander-Tetri BA, Bacon BR (1999). Nonalcoholic steatohepatitis: a proposal for grading and staging the histological lesions. Am J Gastroenterol.

