# Supplementary figures and images for: Altered expression of cell cycle and apoptotic proteins in chronic hepatitis C virus infection
Source: BMC Microbiol. 2008 Aug 5;8:133. doi: 10.1186/1471-2180-8-133 (PMC2518161; doi:10.1186/1471-2180-8-133)

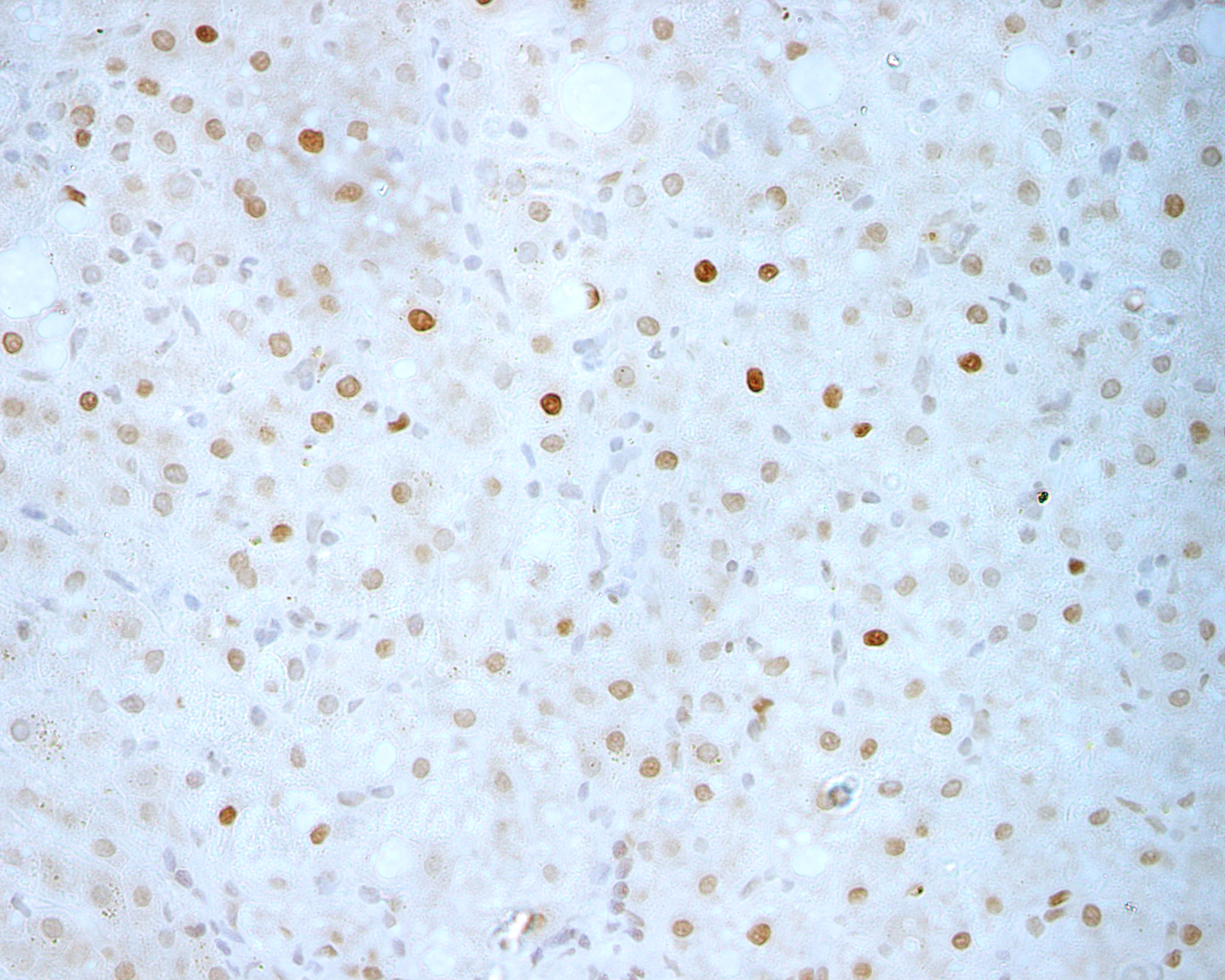

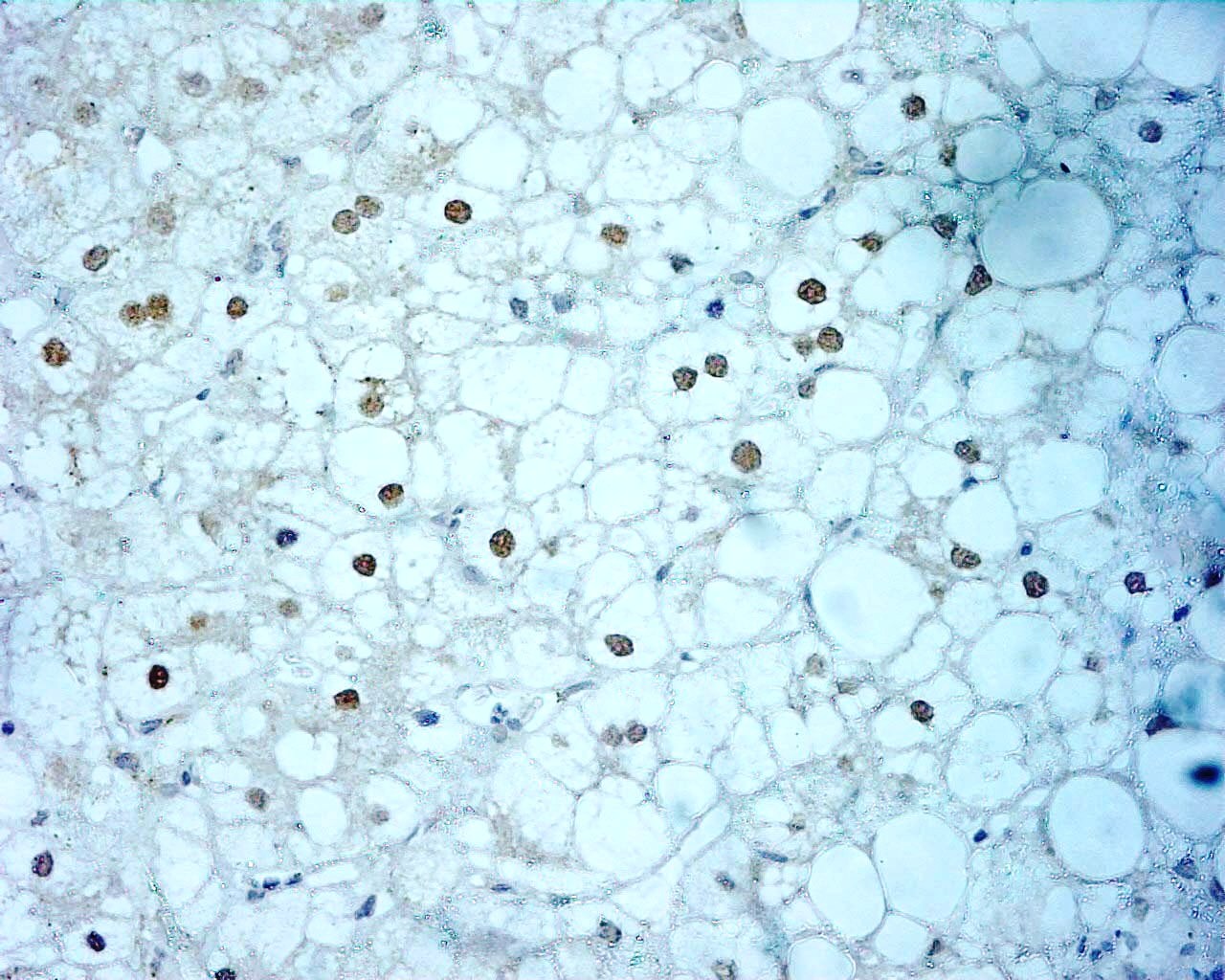


a) NL

b) NL

c) NL

**a) HCV**

**b) HCV**


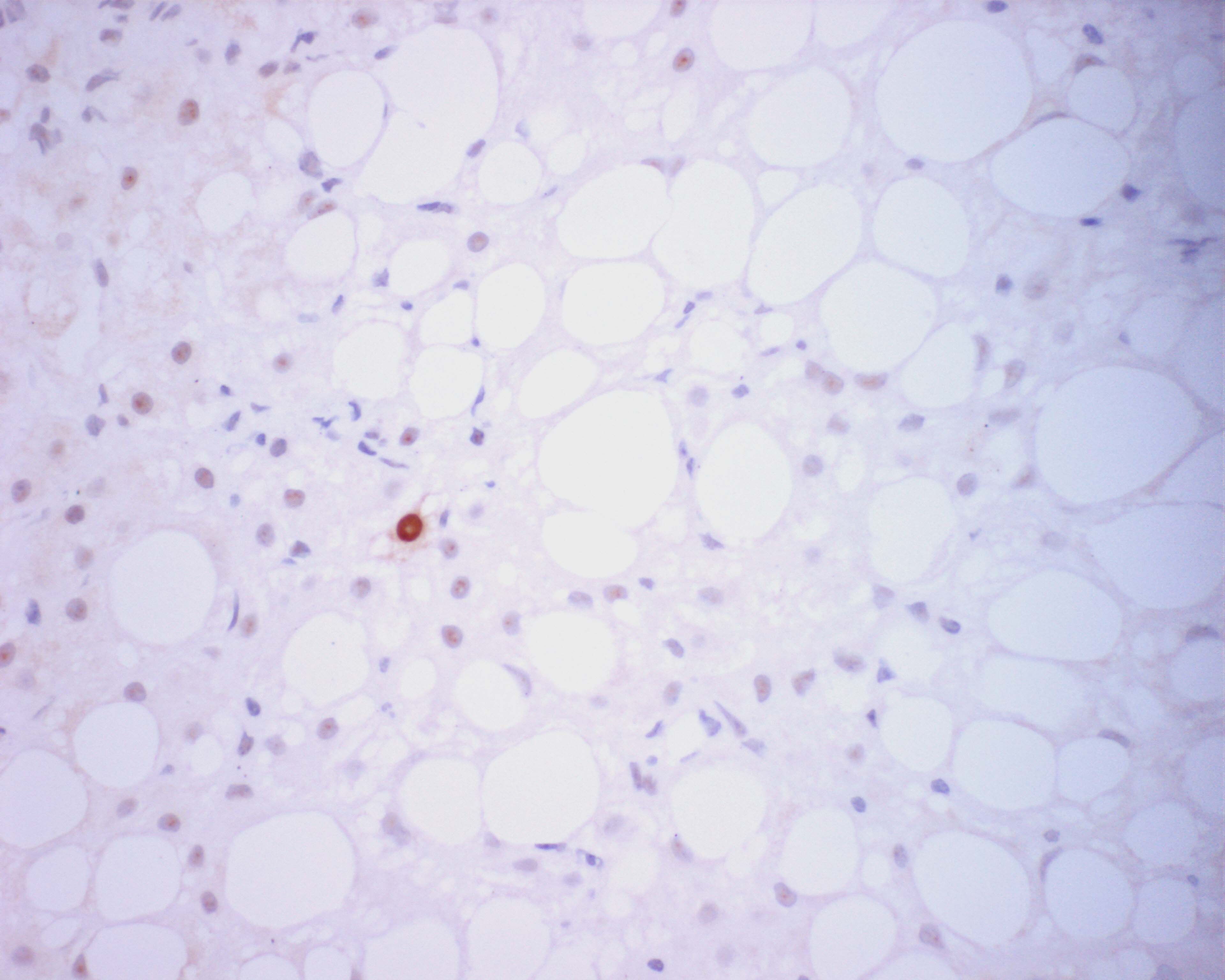


**c) HCV**

Supplement: Additional file 1 — Expression of Mcm-2 and G1-S phase Cyclins. Immunohistochemical staining of biopsy specimens from HCV-infected patients (HCV). Sections were stained using a) anti-Mcm-2, b) anti-Cyclin D and c) anti-Cyclin A. Positive hepatocytes are stained brown (Mayer hematoxylin, magnification 400×). [file 1471-2180-8-133-S1.doc]
